# Pilot Trial of Arginine Deprivation Plus Nivolumab and Ipilimumab in Patients with Metastatic Uveal Melanoma

**DOI:** 10.3390/cancers14112638

**Published:** 2022-05-26

**Authors:** Lukas Kraehenbuehl, Aliya Holland, Emma Armstrong, Sirinya O’Shea, Levi Mangarin, Sara Chekalil, Amanda Johnston, John S. Bomalaski, Joseph P. Erinjeri, Christopher A. Barker, Jasmine H. Francis, Jedd D. Wolchok, Taha Merghoub, Alexander N. Shoushtari

**Affiliations:** 1 Ludwig Collaborative and Swim Across America Laboratory, Parker Institute for Cancer Immunotherapy, Human Oncology and Pathogenesis Program, Department of Medicine, Memorial Sloan Kettering Cancer Center (MSKCC), New York, NY 10065, USA; kraehenl@mskcc.org (L.K.); hollanda@mskcc.org (A.H.); mangaril@mskcc.org (L.M.); chekalis@mskcc.org (S.C.); wolchokj@mskcc.org (J.D.W.); merghout@mskcc.org (T.M.); 2 Department of Medicine, Memorial Sloan Kettering Cancer Center (MSKCC), New York, NY 10065, USA; armstroe@mskcc.org (E.A.); osheas@mskcc.org (S.O.); 3 Polaris Pharmaceuticals, Inc., San Diego, CA 92121, USA; ajohnston@polarispharma.com (A.J.); jbomalaski@polarispharma.com (J.S.B.); 4 Department of Radiology, Memorial Sloan Kettering Cancer Center (MSKCC), New York, NY 10065, USA; erinjerj@mskcc.org; 5 Department of Radiation Oncology, Memorial Sloan Kettering Cancer Center (MSKCC), New York, NY 10065, USA; barkerc@mskcc.org; 6 Ophthalmic Oncology Service, Department of Surgery, Memorial Sloan Kettering Cancer Center (MSKCC), New York, NY 10065, USA; francij1@mskcc.org; 7 Weill Cornell Medical College; New York, NY 10065, USA

**Keywords:** uveal melanoma, immunotherapy, arginine depletion, ADI-PEG 20

## Abstract

**Simple Summary:**

Uveal melanoma is a rare subtype of malignant melanoma. It is known to rapidly metastasize, with the liver being the most frequently affected organ. Due to differences from melanoma arising in the skin, such as a lower number of mutations, it responds poorly to immune checkpoint blockade, a treatment approach reinvigorating the patient’s immune system to eliminate the cancer. We here investigated the safety and tolerability of a new combination treatment consisting of two established immunotherapy medications (ipilimumab and nivolumab) with the addition of an experimental arginine depleting medication, pegylated arginine deiminase (ADI-PEG 20), which is thought to make uveal melanoma more amenable to immunotherapy. This novel treatment approach was found to be safe and well-tolerated but did not improve the clinical outcome beyond the expected limited efficacy of approved immunotherapy alone.

**Abstract:**

Metastatic uveal melanoma (UM) remains challenging to treat, with objective response rates to immune checkpoint blockade (ICB) being much lower than in primary cutaneous melanoma (CM). Besides a lower mutational burden, the overall immune-excluded tumor microenvironment of UM might contribute to the poor response rate. We therefore aimed at targeting deficiency in argininosuccinate synthase 1, which is a key metabolic feature of UM. This study aims at investigating the safety and tolerability of a triple combination consisting of ipilimumab and nivolumab immunotherapy and the metabolic therapy, ADI-PEG 20. Nine patients were enrolled in this pilot study. The combination therapy was safe and tolerable with an absence of immune-related adverse events (irAE) of special interest, but with four of nine patients experiencing a CTCAE grade 3 AE. No objective responses were observed. All except one patient developed anti-drug antibodies (ADA) within a month of the treatment initiation and therefore did not maintain arginine depletion. Further, an IFNg-dependent inflammatory signature was observed in metastatic lesions in patients pre-treated with ICB compared with patients with no pretreatment. Multiplex immunohistochemistry demonstrated variable presence of tumor infiltrating CD8 lymphocytes and PD-L1 expression at the baseline in metastases.

## 1. Introduction

Patients with advanced uveal melanoma (UM) generally have few effective systemic therapy options. The combination checkpoint blockade with nivolumab plus ipilimumab has a significantly lower objective response rate in UM than in cutaneous melanoma (12–18% versus 60%) [1,2,3,4]. A systematic literature review identified response rates of as little as 0–6.5% (ipilimumab 10 mg/kg), 30% (pembrolizumab) and 6% (nivolumab) [5]. Furthermore, whereas 42–45% of primary cutaneous melanomas carry a BRAF V600E mutation and are therefore predicted to respond to targeted BRAF-/MEK inhibition [6], UM do not carry BRAF V600E mutations [7]. Recently, tebentafusp, an HLA-A specific T-cell redirecting agent targeting the melanocytic protein gp100, was demonstrated to improve the overall survival over pembrolizumab or ipilimumab or dacarbazine, which are relatively suboptimal “standard” choices, in patients with metastatic UM who had an HLA-A 02:01 allele [8,9]. Unfortunately, owing to the restricted HLA background, only half of all patients will ever have this therapeutic option, and the vast majority of patients are still likely to succumb to this disease even with the availability of tebentafusp. To overcome this extensive unmet therapeutic need, an improved understanding of resistance to immune-based therapy in metastatic UM is required.

One hypothesis for the relative lack of efficacy of standard immune checkpoint inhibition is thought to be related to the lower median tumor mutational burden (TMB) of UM versus cutaneous melanoma and its relatively “cold,” non-T-cell inflamed tumor microenvironment (TME) [10,11,12]. However, much of these analyses of uveal melanoma TME have been performed on primary tumors characterized by The Cancer Genome Atlas. Less is known about the TME of metastatic UM. Two recent studies of metastatic UM samples used single cell sequencing and multiplex immunohistochemistry to identify that they do harbor diverse immune infiltrates [13,14]. This suggests that the lack of efficacy may be due to other unique features associated with metastatic UM when compared to cutaneous melanoma, such as a higher rate of exhausted CD8 lymphocytes as a proportion of total CD8 infiltrates [15].

One unique metabolic feature of UM versus cutaneous melanoma is its arginine dependence. Arginine is not an essential amino acid for most cells in the body because it is synthesized from the urea cycle via citrulline using arginosuccinate synthase (ASS) [16]. Many tumor cells, including almost all UM tumors, however, are ASS deficient [17], and for these cells, arginine is essential for nucleotide synthesis. This suggests arginine degrading drugs can be an effective tumor-specific therapy that could be relatively well tolerated. Indeed, a clinical trial of the arginine deiminase ADI PEG 20 showed a modest clinical benefit as monotherapy in UM, with four of six patients with advanced UM in a larger trial demonstrating stable disease for a maximum of 8 months [17].

Clinical data in other tumor types suggest that ADI PEG 20 treatment is associated with an increase in T cell infiltration and elevated PD-L1 expression in the TME. In addition, the combination of ADI PEG 20 with pembrolizumab led to objective responses in 6 of 25 patients (24%) with preceding treatment lines, including 1 partial response in a patient with PD-1 resistant mucosal melanoma [18]. In that trial, CD3+ cell infiltration in tumors during treatment increased in 10 of 12 patients in the study. However, no patients with uveal melanoma were enrolled on the trial.

Building upon the prior activity of ADI PEG 20 in patients with metastatic UM as monotherapy and in parallel with the above combination trial with pembrolizumab, we launched a Phase 1 study of combination ADI PEG 20 with nivolumab + ipilimumab for patients with metastatic UM.

## 2. Materials and Methods

Between May and October 2019, 9 patients with biopsy-confirmed metastatic melanoma regardless of prior therapy were enrolled to this pilot trial to receive ADI PEG 20 36 mg/m^2^ weekly intramuscularly plus nivolumab 240 mg flat dose and ipilimumab 1 mg/kg intravenously every 3 weeks for up to 6 doses followed by nivolumab 480 mg monotherapy as maintenance every 4 weeks. The intent of the nivolumab (aPD1) and ipilimumab (aCTLA4) combination regimen in this trial was to increase the duration of exposure to ipilimumab by reducing the dose and increasing the maximum number of doses from 4 to 6. The primary endpoint was safety, defined by appearance of therapy-associated immune-related adverse events of special interest up to 24 weeks after initial therapy onset as markers of dose limiting toxicity rather than a more typical broad definition of G3 AEs by CTCAE v4.03. These irAEs of special interest included any death (grade 5) event, select grade 4 events such as AST/ALT elevation, pneumonitis, and nephritis; and a composite of select “lower grade” but life-altering events such as grade 2 myocarditis, grade 2 sensory/motor neuropathy, grade 3 bilirubin elevation, or any grade encephalitis. If 3 or more patients experienced an irAE of special interest, the study would be halted, and the combination would be considered intolerable.

Biopsies of UM metastases were obtained at baseline and after 2 weeks of therapy. Plasma levels of arginine and citrulline as well as neutralizing antibodies were tracked over time. Lactate Dehydrogenase and Alkaline phosphatase were measured using standard techniques on day 1 prior to treatment. Patients underwent standard of care tumor genetic testing for GNAQ, GNA11, CYSLTR2, BAP1, SF3B1, and EIF1AX nonsynonymous variants and copy number alterations as part of the MSK-IMPACT multigene sequencing platform, as described previously [19,20].

The Immunofluorescence detections of CD8hu and PDL1hu) were performed at Molecular Cytology Core Facility of the Memorial Sloan Kettering Cancer Center using a Discovery Ultra processor (Ventana Medical Systems. Roche-AZ). After 32 min of heat and CC1 (Cell Conditioning 1, Ventana cat#950-500) retrieval, the tissue sections were blocked first for 30 min in Background Blocking reagent (Innovex, catalog#: NB306). A rabbit monoclonal CD8 antibody (Ventana, cat#790-4460) was used in 0.07 μg/mL concentrations. The incubation with the primary antibody was performed for 2 h, followed by 32 min incubation with biotinylated goat anti-rabbit IgG (Vector labs, cat#:PK6101) in 5.75 μg/mL. The detection was performed with the Secondary Antibody Blocker, Blocker D, Streptavidin-HRP D (Ventana Medical Systems), followed by incubation with a Tyramide-Alexa Fluor 488 (Life Tech, cat#B40932) A rabbit polyclonal PDL1 antibody (Cell Signaling cat #13684) was used in 5 μg/mL concentrations. The incubation with the primary antibody was performed for 6 h, followed by 60 min incubation with biotinylated goat anti-rabbit IgG (Vector labs, cat#:PK6101) in 5.75 μg/mL. The detection was performed with a Secondary Antibody Blocker, Blocker D, Streptavidin-HRP D (Ventana Medical Systems), followed by incubation with Tyramide-Alexa Fluor 488 (Life Tech, cat#B40932). All slides were counterstained in 5 μg/mL DAPI [dihydrochloride 2-(4-Amidinophenyl)-6-indolecarbamidine dihydrochloride], Sigma D9542, for 5 min at room temperature, mounted with anti-fade mounting medium Mowiol [Mowiol 4-88 (CALBIOCHEM code: 475904)] and coverslipped. The immunofluorescence (IF) results were derived using the image analysis platform HALO (version 2.3, Indica Labs, Albuquerque, NM, USA). Nuclear segmentation parameters were set using DAPI as the reference channel, then an analysis mask was overlayed on the image, allowing the software to locate and segment each cell. Using a combination of the analysis mask and the scanned slide image, a minimum intensity threshold was set for each of the images. Following the setting of thresholds, the analysis outputs a percentage of positive cells based on the number of positive cells and the total number of cells.

Bulk RNA sequencing data for each sample was aligned to the Genome Reference Consortium Human Build 37 (GRCh37.75) with STAR (v2.6.0) (Dobin Lab, CSHL, Cold Spring Harbor, NY, USA) [21], available by the gene expression omnibus (GEO) accession number GSE202687. Count-based expression profiles were quantified using Subread featureCounts (v1.5.1) (Shi Lab ONJCRI, Heidelberg, Australia) [22]. Downstream statistics and figure generation was performed on the resulting counts matrix within the R statistical platform. Differential expression comparisons were generated using the DESeq2 package with selected genes (*p* < 0.05).

## 3. Results

### 3.1. Safety and Efficacy

Nine patients were consented, enrolled, and treated (67% female) with a median age of 56 (range: 45–76). Five patients had received prior systemic therapy and four had no prior systemic therapy. All five subjects with prior treatment had received checkpoint inhibitors, and two had received prior tebentafusp on a clinical trial. Two patients previously treated had also received hepatic arterial embolization. See Table 1 for demographics.

There were no irAEs of special interest among the nine patients, and the combination was declared tolerable. Four of nine patients (44%) experienced a grade 3 AE related to therapy (neutropenia, lymphopenia, fever, and vasculitis). There were no Grade 4 or Grade 5 events. Three of nine (33%) had a serious adverse event related to therapy. Patient 1 had a grade 3 fever on day 12 of cycle 1, possibly related to all three study drugs. Patient 2 was hospitalized for the monitoring of grade 2 tachycardia 1 month into therapy, possibly related to ADI PEG 20 and nivolumab. Patient 9 was hospitalized with grade 3 medium-vessel vasculitis (most consistent with polyarteritis nodosa) on day 13 of cycle 1, responsive to a 1 mg/kg prednisone equivalent. Notably, the patient was successfully rechallenged with triplet therapy 4 weeks later and did not experience recurrent vasculitis. No patients stopped study therapy for toxicity. See Table 2 for a detailed list of AEs possibly related to study therapy.

**Figure 1:**
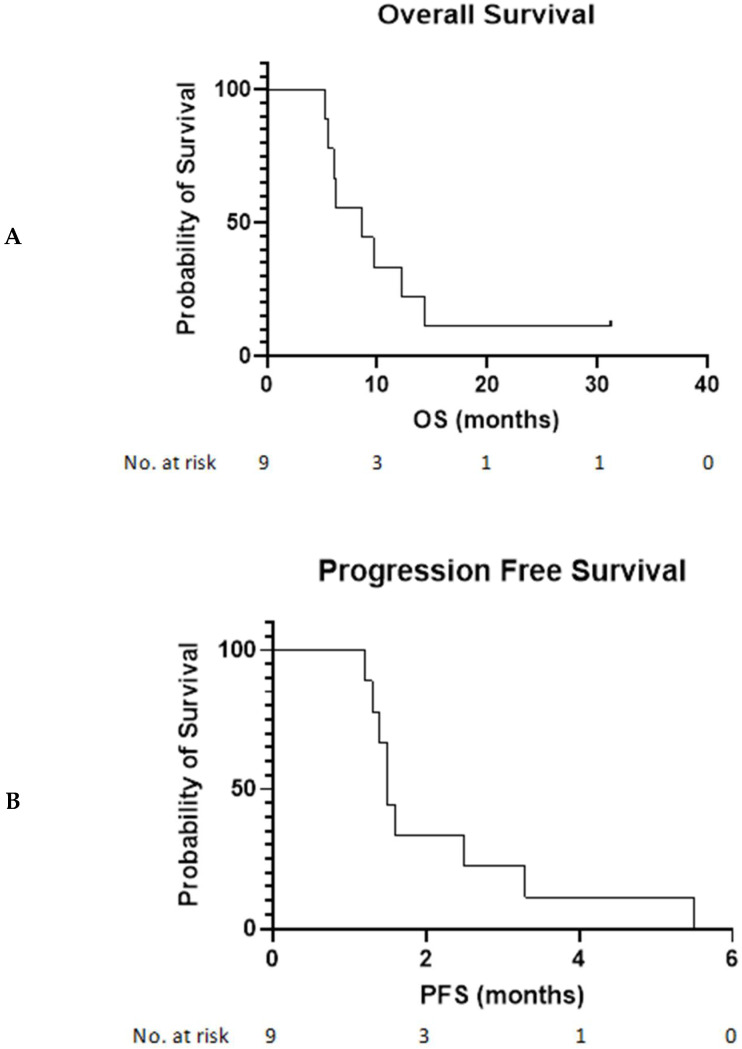
Kaplan–Meier curve displaying limited overall survival in all patients. (**A**) OS = overall survival; (**B**) PFS = progression free survival.

The RECIST 1.1 ORR was 0%, with two of nine patients achieving stable disease. No patients experienced immune-related stable disease by iRECIST. All patients have experienced RECIST progression, and the median PFS was 1.5 months (range: 1.2–5.5 months). The median OS was 8.6 months (range: 5.6–31+ months; see Figure 1). One patient with stable disease experienced subjective clinical benefit, with a reduction in bone pain while on treatment, an objective drop in lactate dehydrogenase (LDH) from 1204 (4.8× upper limit of normal) at baseline to a nadir of 340 (1.8× upper limit of normal) after 3 weeks, and stable disease at week 12. This patient was eventually treated past progression and stopped the study at week 36 for persistent non-target progression. Detailed demographics and toxicity information are included as Supplemental Table S1.

### 3.2. Pharmacodynamics

**Figure 2:**
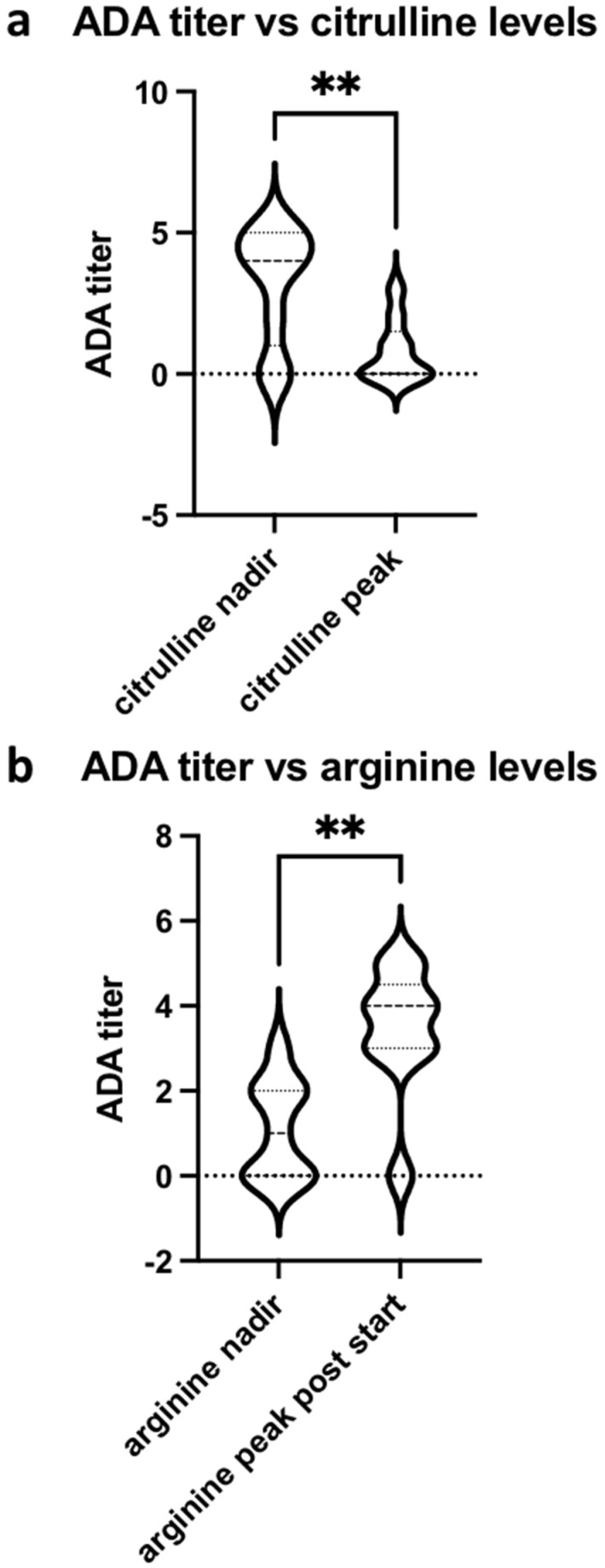
(**a**) Anti-drug antibody titers inversely correlate with serum citrulline. Mean titers are significantly higher when citrulline levels are lowest compared to peak citrulline levels. ADA = anti-drug antibodies, ** = *p* < 0.005; (**b**) Serum arginine correlates with anti-drug antibodies, illustrated by significantly higher titers measured at time of peak serum arginine compared with lowest arginine level. ADA = anti-drug antibodies, ** = *p* < 0.005.

**Figure 3:**
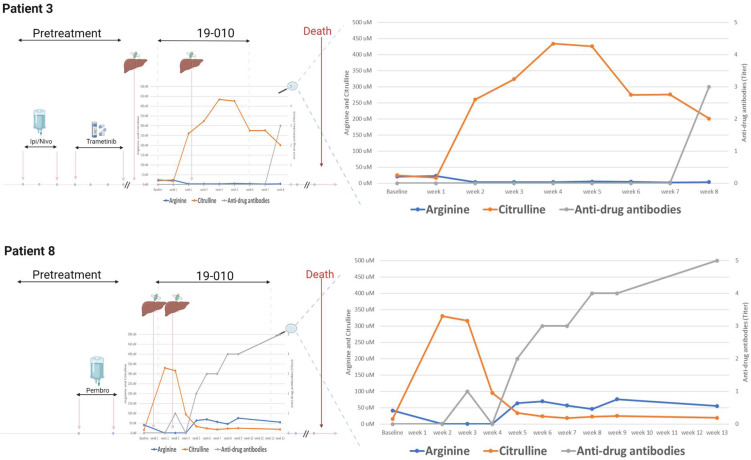
Patient timelines and serologies. Treatment lines prior to study enrolment (left) and serologies (citrulline = orange, arginine = blue, in µM, left *Y*-axis; anti-drug antibody titers = grey, right Y axis). Timepoints of biopsies from liver metastases are illustrated to the left. Pembro: pembrolizumab, Ipi/Nivo: combined ipilimumab and nivolumab.

As expected, serum levels of arginine are inversely correlated with citrulline (r = −0.57, 95% CI −0.6920–−0.42, *p* < 0.0001; Figure 2a). Furthermore, anti-ADI PEG-antibodies titer correlate positively with arginine levels (r = 0.40, 95% CI 0.22–0.56, *p* < 0.0001). Conversely, serum arginine correlates with anti-drug antibodies (ADA, Figure 2b). Plasma arginine levels were reduced to undetectable in all patients by week 2 but by weeks 4–5 had recovered to near baseline levels in eight of nine patients (Figure 3 and Supplementary Figure S1). Notably, patient 3 being the sole participant with delayed development of ADA, the patient achieved RECIST stable disease at the first staging 7 weeks into treatment but came off the study for clinical signs of progression.

### 3.3. Histopathological Assessment of Tumor Biopsies

**Figure 4:**
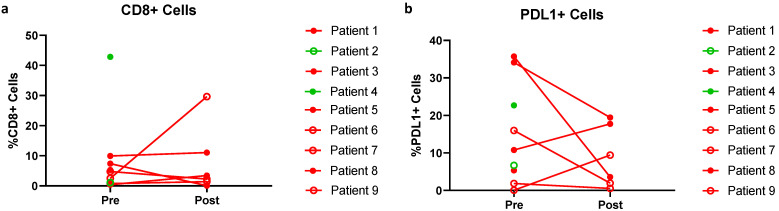
Proportion of CD8 T cells out of all DAPI+ (**a**) and PD-L1+ cells out of all DAPI+ cells (**b**). Green: Patients achieving stable disease at 3 months, red: patients with progression of disease, filled circles: patients with pre-treatment, and empty circles: patients without pre-treatment.

All nine patients had available baseline biopsies; five had week 2 biopsies and one had week 12 post-progression biopsies available. These pairs were analyzed for the presence of CD8+ and PD-L1+ cells by immunofluorescence. Quantifying CD8+ cells out of all (DAPI+) cells, we observed variable trends in CD8 positivity during the treatment course: a more than two-fold increase in two patients, a more than two-fold decrease in two patients and stable CD8 positivity in two patients (Figure 4).

In contrast to prior reports of pre-treatment metastases or primary tumors, the evaluation of PD-L1 expression in these baseline metastatic UM lesions (eight of nine of which were from the liver) demonstrated a broad range of positivity (0.04–35.73%). In addition, there was no statistically significant change in PD-L1 expression level from the baseline compared to the on-treatment time point in the six paired biopsies. In four patients, a more than two-fold decrease in PD-L1 level was observed, whereas a more than two-fold PD-L1 increase was found in one, and three patients demonstrated stable proportions of PD-L1 positive cells (Figure 4). Patient 3, the patient maintaining arginine suppression, demonstrated a strong increase in CD8+ cells and decreased PD-L1 expression at follow up.

### 3.4. Bulk RNA Sequencing of Baseline Tumor Biopsies

**Figure 5:**
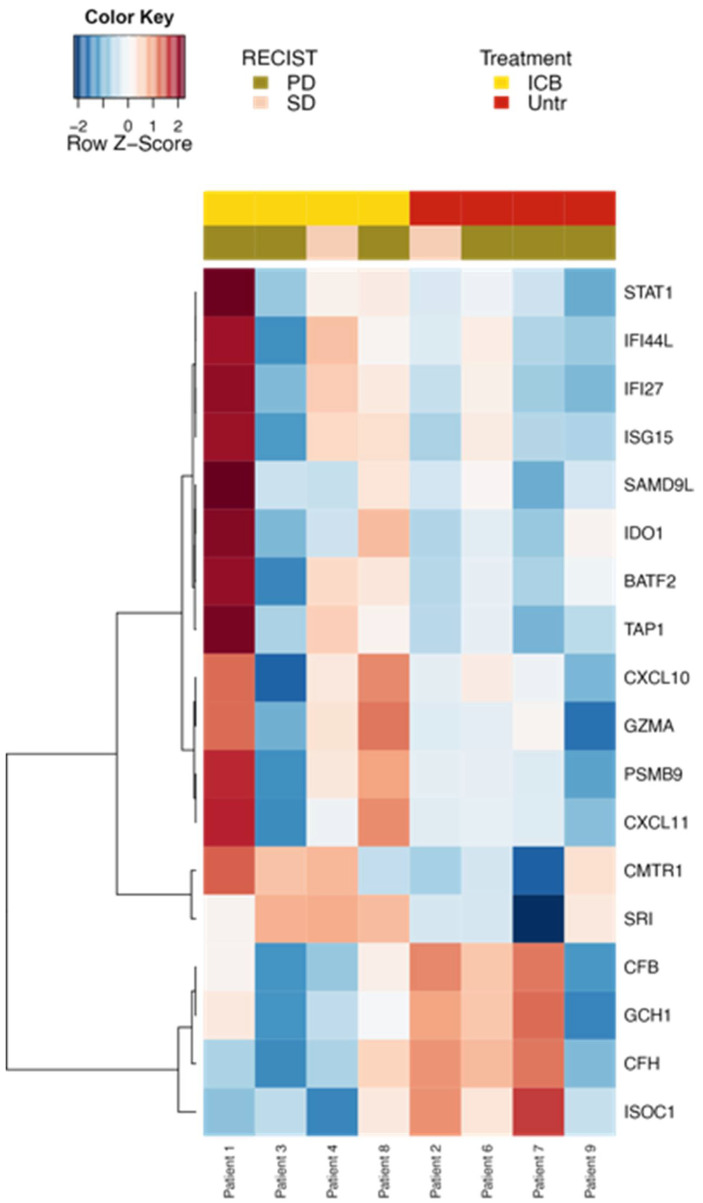
RNA expression of genes regulated by interferon gamma is upregulated in patients with preceding ICB compared to untreated patients. Yellow = prior immune checkpoint blockade, red = no pre-treatment, brown = progressive disease at 3 months, and beige = stable disease at 3 months.

Bulk RNA sequencing of baseline samples identified increased expression of interferon gamma-dependent genes when comparing patients treated with prior immune checkpoint blockade (including pembrolizumab monotherapy and combined ipilimumab and nivolumab) versus those with no prior therapy upon trial enrollment. These genes include indoleamine 2,3-deoxygenase 1 and granzyme a, overall representing a strong interferon gamma inflammation signature (Figure 5).

## 4. Discussion

In this pilot trial of arginine degradation with ADI PEG 20 plus combination checkpoint inhibition with nivolumab plus ipilimumab, we utilized a unique definition of immune-related adverse events of special interest to show that this triplet therapy was safe and tolerable. This is a useful paradigm for future triplet therapies building on nivolumab plus ipilimumab, which has a very high rate of grade 3 or higher adverse events using CTCAE version 4. Simply defining the tolerability by CTCAE grade 3 or higher adverse events is unlikely to accurately depict the tolerance of this combination, since many higher-grade AEs are reversible (e.g., isolated AST/ALT elevations) and yet certain lower grade AEs are permanent or debilitating (e.g., neuropathy). We have defined immune-related adverse events of special interest to encompass the most clinically relevant irAEs, regardless of grade (e.g., high grade colitis and hepatitis, but lower grade neurotoxicity and any grade myocarditis) and incorporated ADI PEG 20-specific adverse events such as anaphylaxis to create a study-specific measure of toxicity. Thus, while the G3 rate of 44% was reasonably high, we demonstrated that patients can tolerate the triplet therapy. In fact, two of the four patients experienced G3 laboratory findings only.

Unfortunately, this triplet therapy did not lead to any objective responses, and only two patients achieved stable disease at 3 months. Several factors might have contributed to this unfavorable outcome. Most importantly, this cohort of patients was at a high risk of progression. Five of nine patients had been previously treated with the PD-1 immune checkpoint blockade, including four with nivolumab plus ipilimumab, two with prior tebentafusp, and two with prior hepatic embolization. Among the four patients with no prior treatment, two of four had an elevated baseline LDH. Overall, this ORR is unlikely to represent a “worse” efficacy than the published ORRs in larger phase 2 trials of 12–18% [1,2]. The disease control rate at 12 weeks was 22% (95% CI 3–60%), which overlaps with the 95% confidence interval of the larger trials [1,2].

Our correlative analyses in plasma suggest one mechanism of progression may be the rapid development of anti-ADI PEG 20 antibodies and reversion of arginine suppression to pre-treatment baseline levels after 1 month of therapy. This is in contrast to the experience of ADI PEG 20 combined with cisplatin and pemetrexed in mesothelioma [23] and with gemcitabine and docetaxel in leiomyosarcomas [24], where the combination has an established response rate. In those latter two trials, chemotherapy may have suppressed the development of antibodies against ADI PEG 20 and enhanced synergy, whereas in this trial immune checkpoint inhibitors may have spurred their development and contributed to the neutralization of a potential benefit of ADI PEG 20. One patient (patient 3) maintained arginine depletion due to a delayed development of ADA. This patient also had a brisk increase in CD8+ T cells and a decrease in PD-L1 expression. However, there was no clinical benefit at 3 months, the patient withdrew from the study due to progression.

The lack of increasing tumor infiltrating CD8 T-cells as well as PD-L1 expression can both represent a lack of therapeutic efficacy or a sampling error due to the nature of the biopsies (ultra-sound-guided core needle).

Subsequent clinical trials of combined arginine depletion with immune checkpoint blockade should aim at overcoming the rapid development of ADA through optimized timing, alternative drug delivery modes, or development of a less immunogenic compound. A previous investigation comparing the immune infiltrate in uveal versus primary cutaneous melanoma identified comparable amounts of CD8 tumor infiltrating lymphocytes in both groups but significantly lower PD-L1 expression in uveal melanoma [25]. We found that PD-L1 expression is variable between tumors but can be elevated in metastatic UM. Along the suggestion of Rossi and colleagues, UM might be immune-privileged at the site of the primary tumor due to the blood–brain barrier, whereas metastatic lesions might be more accessible to immune cells and therefore novel treatments that target checkpoints beyond PD-1/L1 and CTLA-4 [26].

Further ongoing clinical trials aiming at enhancing the efficacy of ICB in melanoma include combination with local radiation [27], additional intrathecal administration of ICB [28], or a histone deacetylase inhibitor [29], amongst others [30]. In addition, a trial combining the anti-LAG3 antibody relatlimab with nivolumab is promising [31]. This combination has now been approved for use in unresectable or metastatic melanoma. The approval is based on improved PFS but not OS in a pivotal trial excluding uveal melanoma [32].

Our correlative analyses nonetheless provide insights that are applicable to other studies with ADI PEG 20 and in uveal melanoma. Most importantly, we demonstrate that in contrast to the primary uveal melanoma cohort analyzed in detail by the TCGA, metastatic uveal melanomas do not uniformly lack PD-L1 expression. This challenges the notion that these tumors are “cold, immune deserts” and is consistent with recent publications of single-cell sequencing of metastatic tumors, demonstrating that checkpoints such as LAG-3 are present in the majority of these tumors [13,14]. Metastatic UM samples exposed to prior immune checkpoint inhibition appeared to display an interferon gamma-dependent inflammation signature compared to UM samples that were previously untreated. Importantly, this also suggests that one cannot simply use the induction of interferon gamma or PD-L1 expression as a surrogate for clinical efficacy of immunotherapy in this disease. This has potential implications for future clinical trial design in UM. The prior receipt of immune checkpoint inhibition treatments should be noted and evaluated to better understand the context of future reports.

## 5. Conclusions

In summary, in this pilot study in a high-risk cohort of nine patients with metastatic uveal melanoma, treatment with ADI PEG 20 plus nivolumab and ipilimumab was safe and tolerable, but it did not lead to major clinical benefit. This may be partially related to the rapid development of ADAs in most patients. Correlative analyses suggest the immune microenvironment in metastatic uveal melanoma is heterogeneous and not the “immune desert” as suggested by earlier studies.

## Figures and Tables

**Table 1:** Demographics.

	Part 1 (n = 9)
**Median Age** years (range)	56 (45–76)
**Sex** (F/M)	6/3
**AJCC Uveal Melanoma Stage**	
M1a	2 (22%)
M1b	6 (67%)
M1c	1 (11%)
**Prior Systemic Therapy**	
No	4 (45%)
Yes	5 (55%)
Nivolumab + Ipilimumab	4 (45%)
PD-1 monotherapy	4 (45%)
Tebentafusp	2 (22%)
**Prior Hepatic Embolization**	2 (22%)
**LDH**	
Elevated	5 (56%)
Normal	4 (44%)
**Alkaline Phosphatase**	
Elevated	4 (44%)
Normal	5 (56%)
**Primary Alterations**	
GNAQ Q209x	2
GNA11 Q209x	7
**Secondary Alterations**	
BAP1	3
SF3B1	4
Wild-type for above	2

**Table 2:** All toxicities at least possibly related to one of the study drugs.

	ADI PEG 20				Nivolumab				Ipilimumab			
	Any	G1-G2	G3	% of Total	Any	#G1-2	#G3	% of Total	Any	#G1-2	#G3	% of Total
Arthralgias/arthritis	7	7	0	78	7	7	0	78	6	6	0	67
AST/ALT increased	6	6	0	67	6	6	0	67	4	4	0	44
Rash	6	6	0	67	6	6	0	67	6	6	0	67
Anemia	5	5	0	56	5	5	0	56	5	5	0	56
Nausea	5	5	0	56	5	5	0	56	4	4	0	44
Alk phos increased	4	4	0	44	4	4	0	44	3	3	0	33
Hypoalbuminemia	4	4	0	44	4	4	0	44	4	4	0	44
Pruritus	4	4	0	44	4	4	0	44	4	4	0	44
Fever	3	2	1	33	3	2	1	33	2	1	1	22
Thrombocytopenia	3	3	0	33	3	3	0	33	3	3	0	33
Vomiting	3	3	0	33	3	3	0	33	3	3	0	33
Leukopenia	3	3	0	33	3	3	0	33	3	3	0	33
Hot flashes	2	2	0	22	2	2	0	22	2	2	0	22
Hyperuricemia	2	2	0	22	2	2	0	22	2	2	0	22
Injection site reaction	2	2	0	22	0	0	0	0	0	0	0	0
Lymphopenia	2	1	1	22	2	1	1	22	2	1	1	22
Neutropenia	2	1	1	22	1	0	1	11	1	0	1	11
Anorexia	1	1	0	11	1	1	0	11	1	1	0	11
Back Pain	1	1	0	11	1	1	0	11	1	1	0	11
Belching	1	1	0	11	1	1	0	11	1	1	0	11
Elevated troponin	1	1	0	11	2	2	0	22	1	1	0	11
Diarrhea	1	1	0	11	2	2	0	22	1	1	0	11
Edema limbs	1	1	0	11	1	1	0	11	1	1	0	11
Fatigue	1	1	0	11	1	1	0	11	0	0	0	0
GERD	1	1	0	11	1	1	0	11	0	0	0	0
Hypocalcemia	1	1	0	11	1	1	0	11	1	1	0	11
Hyponatremia	1	1	0	11	1	1	0	11	1	1	0	11
Hypothyroidism	1	1	0	11	1	1	0	11	1	1	0	11
Insomnia	1	1	0	11	1	1	0	11	0	0	0	0
Edema, localized	1	1	0	11	1	1	0	11	1	1	0	11
Nasal congestion	1	1	0	11	1	1	0	11	0	0	0	0
Pain	1	1	0	11	1	1	0	11	1	1	0	11
Cough	1	1	0	11	1	1	0	11	1	1	0	11
Sinus tachycardia	1	1	0	11	1	1	0	11	1	1	0	11
Vasculitis	1	0	1	11	1	0	1	11	1	0	1	11
Dry mouth	0	0	0	0	1	1	0	11	1	1	0	11
Dyspepsia	0	0	0	0	1	1	0	11	0	0	0	0

G = CTCAE grade, AST = Aspartate transaminase, ALT = Alanine transaminase, Alk Phos = Alkaline phosphatase, GERD = gastroesophageal reflux disease.

## Data Availability

Fully anonymized data are available on request.

## Supplementary Materials

The following supporting information can be downloaded at: https://www.mdpi.com/article/10.3390/cancers14112638/s1, Figure S1: Additional patient timelines and serologies; Table S1: Additional clinical information.
