# Supplementary material for: Pilot Trial of Arginine Deprivation Plus Nivolumab and Ipilimumab in Patients with Metastatic Uveal Melanoma
Source: Cancers (Basel). 2022 May 26;14(11):2638. doi: 10.3390/cancers14112638 (PMC9179243; doi:10.3390/cancers14112638)
Supplement: Supplementary file 1 [file cancers-14-02638-s001.zip › Figure S1.pdf]

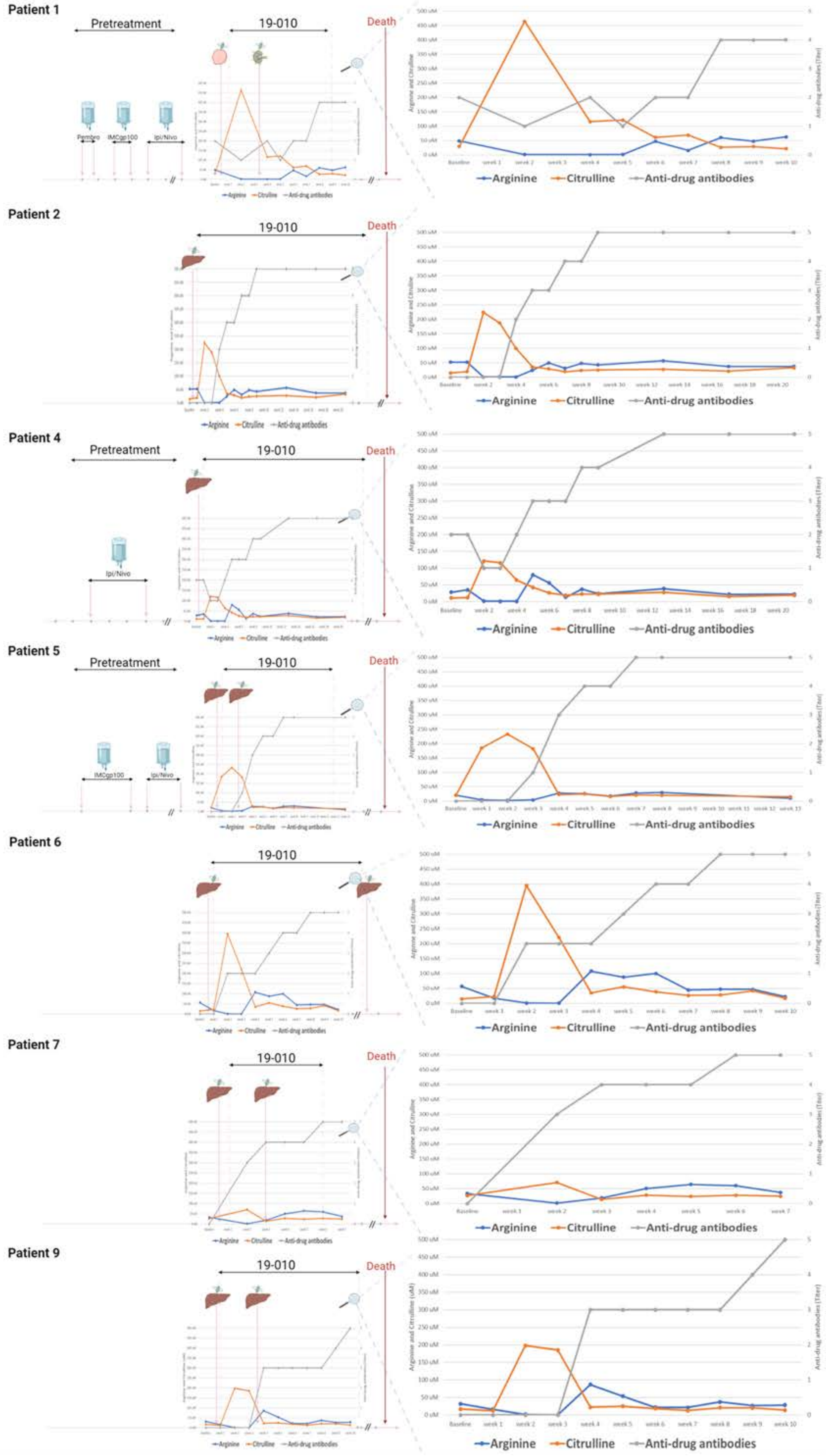

Patient 1
Pretreatment
19-010
Death
Pembro
IMCgp100
Ipi/Nivo
Arginine and Citrulline
Anti-drug antibodies (Titer)
Arginine
Citrulline
Anti-drug antibodies
Patient 2
19-010
Death
Arginine
Citrulline
Anti-drug antibodies
Patient 4
Pretreatment
19-010
Death
Ipi/Nivo
Arginine
Citrulline
Anti-drug antibodies
Patient 5
Pretreatment
19-010
Death
IMCgp100
Ipi/Nivo
Arginine
Citrulline
Anti-drug antibodies
Patient 6
19-010
Arginine
Citrulline
Anti-drug antibodies
Patient 7
19-010
Death
Arginine
Citrulline
Anti-drug antibodies
Patient 9
19-010
Death
Arginine and Citrulline (uM)
Arginine
Citrulline
Anti-drug antibodies

Figure S1. Additional patient timelines and serologies. Timepoints of biopsies of metastases in subcutaneous nodule (Pt 1), lymph node (Pt 1) and liver (other patients) are noted to the left. Treatment lines prior to study enrolment (left) and serologies magnified to the right (citrulline = orange, arginine = blue, in μM, left y-axis; anti-drug antibody titers = grey, right y axis). Pembro: pembrolizumab, Ipi/Nivo: combined ipilimumab and nivolumab, IMCgp100: tebentafusp.

Table S1. Additional clinical information including age, sex, stage, liver- and non-liver metastases, type of prior systemic therapy, liver embolization, LDH- and Alkaline Phosphatase elevation, genetic alterations, RECIST at 12 weeks, outcome, dosing, related- and unrelated adverse events. Met = metastasis, embo = embolization, LDH = lactate dehydrogenase, alk phos = alkaline phosphatase, RECIST = response evaluation criteria in solid tumors, PFS = progression free survival, OS = overall survival, mo = months
